# Bacterial associations reveal spatial population dynamics in *Anopheles gambiae* mosquitoes

**DOI:** 10.1038/srep22806

**Published:** 2016-03-10

**Authors:** Moritz Buck, Louise K. J. Nilsson, Carl Brunius, Roch K. Dabiré, Richard Hopkins, Olle Terenius

**Affiliations:** 1 Evolutionary Biology Center and Bioinformatics Infrastructure for Life Sciences, Uppsala University, Norbyvägen 18D, 752 36 Uppsala Sweden; 2 Department of Ecology, Swedish University of Agricultural Sciences (SLU), 750 07 Uppsala, Sweden; 3 Department of Food Science, Swedish University of Agricultural Sciences (SLU), 750 07 Uppsala, Sweden; 4 Institut de Recherche en Sciences de la Santé/Centre Muraz, O1 BP 390 Bobo-Dioulasso 01, Burkina Faso; 5 Natural Resources Institute, University of Greenwich, Central Avenue, Chatham Maritime, Kent, ME4 4TB, United Kingdom

## Abstract

The intolerable burden of malaria has for too long plagued humanity and the prospect of eradicating malaria is an optimistic, but reachable, target in the 21^st^ century. However, extensive knowledge is needed about the spatial structure of mosquito populations in order to develop effective interventions against malaria transmission. We hypothesized that the microbiota associated with a mosquito reflects acquisition of bacteria in different environments. By analyzing the whole-body bacterial flora of *An. gambiae* mosquitoes from Burkina Faso by 16 S amplicon sequencing, we found that the different environments gave each mosquito a specific bacterial profile. In addition, the bacterial profiles provided precise and predicting information on the spatial dynamics of the mosquito population as a whole and showed that the mosquitoes formed clear local populations within a meta-population network. We believe that using microbiotas as proxies for population structures will greatly aid improving the performance of vector interventions around the world.

As a part of a holistic approach to controlling the disease, the eradication of malaria requires a strong intervention against the vectors transmitting malaria. Today, impregnated bednets are efficient barriers of night-time malaria transmission. However, the anthropophilic malaria mosquitoes have shifted their feeding patterns to circumvent the bednet barriers, whilst an increasing pesticide resistance in the mosquitoes also reduces the effectivity of bednets and indoor residual spraying^1^. Under these prevailing conditions it is anticipated that new strategies must be explored which eliminate the parasites in the mosquitoes themselves. Control strategies involving genetic modification of mosquitoes (transgenesis) and genetic modification of their gut bacteria (paratransgenesis) both build on the premises that once released, the modified organism is maintained and spreads by itself through the vector population^2^,^3^. In paratransgenesis, the delivery of modified bacteria can be either through larval breeding sites or artificial sugar sources, but fundamental knowledge of where malaria mosquitoes acquire their bacteria is lacking. For transgenesis, the prerequisite for a successful intervention is good knowledge of malaria mosquito life history including dispersal distances, formation of local- and metapopulations and rates of exchange between populations.

Based on the hypothesis that the microbiota associated with a mosquito can be seen as a shadow cast by life-history events, where different environments leave their mark by contributing to the flora associated with the insect, we collected *An. gambiae* adult mosquitoes from three villages in Burkina Faso (Fig. 1). We analyzed the whole-body bacterial flora of the mosquitoes using 16 S amplicon sequencing yielding an average of 17,300 (SD 13,161.83) sequences per mosquito (see Methods and Supplementary Fig. S1). After selecting the mosquitoes with at least 7500 sequences based on rarefaction-curves analysis (Supplementary Fig. S2), we found that the mosquito samples harbored a wide range of bacterial taxa and display clear individual differences (Fig. 2). Horn clustering analysis of the samples showed that the mosquitoes clustered according to village with very few exceptions (Supplementary Fig. S3). The tightest clustering appeared between female mosquitoes from the same village, but clustering was equally common between gravid and non-blood-fed mosquitoes within those villages. NMDS score plots show apparent clustering of mosquitoes according to where the malaria mosquitoes were captured (Fig. 3), indicating a strong association between the microbiota composition and village of capture. The sub populations in the different villages are clearly separated despite being within flight distance from each other, but a couple of samples from the village VK3 have a bacterial flora more similar to the bacteria associated with mosquitoes of villages VK5 and VK7. This indicates that there is some interaction between the villages and therefore suggests the existence of local populations within a meta-population network.

In a supervised model, random forest classification showed 90% successful classification (i.e. 26 out of 29 individual samples; Fig. 4), thus confirming clustering and association between microbiota and village of capture and furthermore providing powerful predictive capabilities of class (village) assignment based on microbiota composition. Moreover, the use of the rdCV (repeated double cross validation) procedure provides a population of classification probability estimates per observation, providing additional depth of information regarding the certainty of classification compared to point estimates. Aside from the fact that most observations are correctly classified, some observations should be commented upon: i) The majority of samples (22 out of 29) had probability distributions practically without overlap between locations, thus indicating individuals with a full life history of exposure to location-specific microbiota and as a consequence unambiguous prediction results; ii) Two such samples from VK7 (VK7-21 and 22) were clearly classified as VK3. Although the causality of this discrepancy is not known, our hypothesis is that this clear case of misclassification corresponds to individuals from VK3 having recently flown in to VK7, thus not having had the time to acquire the OTU signature of VK7; iii) Four samples (VK3-41 and VK7-28, 31–35 and 36–40) showed substantial overlap between the probability distributions of VK3 and VK7 and three samples (VK3-47 and VK5-2 and 16–20) similarly between all three locations. We hypothesize that these findings relate to individuals either with longer flying distances or older individuals with longer flight history having therefore been exposed to several location-specific microbiota, and; iv) There is no evidence for a difference between males and females in terms of classification accuracy. Although the accuracy of data on males is potentially decreased by the pooling of individuals to ensure a sufficient quantity of DNA, the high correct classification rate indicates such a high degree of location fidelity that pooling from a practical perspective seems to not be an issue. It should be noted that predictive models where the low number of observations for practical purposes do not allow separation of data into training, validation and testing sets are easily over-fitted to spurious “false positive” variables, as is the case for the current data set^4^. However, in the present case, permutation analysis clearly showed both model validity (p = 2.4 * 10^−8^; Supplementary Fig. S4) and that the rdCV procedure effectively minimized statistical overfitting by comparing the estimated H0 mean (19.4 misclassifications) to the predicted H0 mean (i.e. 2/3 * 29 observations ≈ 19.3 misclassifications).

The most important determinants of the random forest classification are in the order of importance the genera *Massilia*, *Wolbachia*, *Shewanella* and *Acinetobacter* (Supplementary Table S1). These four genera are likely acquired at different time points during the mosquito life history. The first time point from which we obtain information is the egg stage. *Wolbachia* is maternally inherited via the egg and was recently discovered for the first time in *An. gambiae* mosquitoes, from Burkina Faso^5^. In our data set, *Wolbachia* displayed a pronounced difference between the villages. It was found in all our specimens from VK5 and in several of the VK5 mosquitoes at a high abundance (36–47%), but in contrast at a low frequency and abundance in both VK3 and VK7 (Fig. 5). The mosquitoes in this study were captured wild in the adult stage; therefore, it is very unlikely that they all stem from a single mother. The predominance for *Wolbachia* in VK5 therefore suggests a high degree of village fidelity that extends back in time to the previous generation. This would imply that effective malaria interventions in one village would not be rapidly undermined by the invasion of malaria mosquitoes from a neighboring village. In the paper by Baldini *et al.*^5^, the *Wolbachia* obtained from *An. gambiae* M and S forms are reported to belong to a novel strain/supergroup of *Wolbachia*. This statement is based on the analysis of concatenated sequences from whole-genome shotgun metagenomic sequencing. For us to be able to compare with their data, we have used the W-spec 16 S sequences found in the supplementary data of Baldini *et al.*^5^ together with our data and previously published sequences. We find that both our W-spec sequences and those of Baldini *et al.*^5^ cluster together with the A and B supergroups (Fig. 6); thus, our analysis does not support the proposition of a new *Anopheles*-specific *Wolbachia* strain. One should note however, that for a final designation of supergroup in *Wolbachia* from the *Anopheles* mosquitoes in Burkina Faso, phylogenetic analyses of sequences from *Wolbachia* wsp or fbpA would be required. Unfortunately, neither we, nor Baldini *et al.*^5^ were able to obtain sequences from these genes and thus the final designation of the *Wolbachia* supergroup will await future work. Our data corroborate only one of the two *Wolbachia* strains found by Baldini *et al.*^5^ (Fig. 6), which could suggest the existence of multiple *Wolbachia* infections, but it would require extensive sampling over time to understand whether indeed two populations of *Wolbachia* are present in the *An. gambiae* M form.

The second time point from which we obtain information is the larval stage. The genera *Massilia* and *Shewanella* possibly reflect larval breeding sites since both *Massilia* and *Shewanella* form biofilms in fresh water^6^,^7^, a common food source for mosquito larvae. Indeed, *Shewanella* was one out of twelve genera isolated from midguts of Iranian *Anopheles* larvae^8^ and was also abundant in egg masses in non-biting midges (Chironomidae), the most common fresh-water insect^9^.

The third time point is connected to the adult food source (for females predominantly during early adulthood). Both male and female mosquitoes use nectar as a sugar source and in the early life as adult mosquitoes both sexes search for nectar sources only. A predominant genus of bacteria in nectar is the *Acinetobacter* where the abundance were between 49% and 90% in tropical plants according to 454 sequencing of 16 S amplicons^10^. Specific analysis of *Acinetobacter* revealed it to be present in almost all mosquitoes with the highest abundance in males (Supplementary Table S2), which is as expected since they only feed on sugar sources. The variable abundance of *Acinetobacter* in different mosquitoes may reflect the time between a nectar meal and capture or the intensity of feeding on a specific flower species. The most abundant *Acinetobacter* species show no difference between males and females, but that the mosquitoes in VK5 share a distinct set of *Acinetobacter* different from what is found in the villages VK3 and VK7 (permutation-based ANOVA: p = 1.7 * 10^−5^; Fig. 7). The data may reflect different nectar sources in the villages since *Acinetobacter* species often are plant-specific^10^,^11^. It also implies that the mosquitoes have predominantly fed on nectar in the vicinity of where they were captured and thus supports the hypothesis of village fidelity suggested by the data obtained from *Wolbachia* sequences.

We have also looked at what could be regarded as the last important life history event for a female mosquito, namely the blood meal. The similarity between gravid and non-blood-fed females in our data set indicates that the bacterial flora associated with the blood-host-seeking experience and blood feeding does not have a major impact on the general microbiota associated with gravid females (Fig. 3). The blood meal, once acquired, leads to rapid proliferation of bacteria^12^,^13^,^14^, but favors a limited number of bacterial species that can utilize the blood and at the same time survive the oxidative stress that follows blood degradation^15^. However, our data show that this rapid shift in gut flora composition is no longer strongly affecting the overall microbiota a few days after the blood meal, indicating that the gut flora may revert to a pre-blood meal composition after the blood meal has been digested. The volatiles produced by skin bacteria play an important role in the guidance of female *An. gambiae* to humans^16^; it is not clear, however, if any bacteria also are picked up from the skin during feeding. We therefore specifically analyzed the presence of the three genera of human skin bacteria most strongly attracting *An. gambiae* females: *Bacillus*, *Brevibacterium* and *Corynebacterium*^16^, but found no correlation to sex/diet (Supplementary Fig. S5).

When analyzing the bacteria on a genus level, we find that the most abundant genera, making up more than 40% of all sequences identified to genus level, are *Thorsellia*, *Wolbachia*, *Massilia* and *Acinetobacter* (Supplementary Table S3). Of these, the most abundant is *Thorsellia*, which was originally isolated in Kenya from *An. gambiae*^17^ and has since then been shown to be the dominant species in different Kenyan populations of *An. gambiae* mosquitoes^15^,^18^. Briones *et al.*^15^ consistently isolated *T. anophelis* from the water surface micro-layer (SML), i.e., where mosquito larvae feed, as well as in 40% of the adults, while Wang *et al.*^15^ found that almost 70% of the bacteria in young adults belonged to *Thorsellia*. The *Thorsellia* sequences obtained in Burkina Faso correspond well to the three species of *Thorsellia* isolated from Kenya^19^,^20^, but also suggest the existence of other *Thorsellia* species (Supplementary Fig. S6).

The rate and direction at which insects move, spread and interact is key information for vector control strategies. From a monitoring point of view it can provide vital information on the movement patterns of individual insects that may carry disease between communities. For control measures aimed at altering the vector competence of insect populations, knowledge about insect movement is a fundamental prerequisite. A recent example is the very successful malaria intervention on the island Zanzibar where intensified usage of impregnated bednets and adequate drug treatment has brought malaria down by 80% and child mortality by 50%^21^. Whether this kind of intervention is also applicable on the mainland depends very much on the dispersal of malaria mosquitoes between communities. Our data suggest that neighboring villages function as metapopulations with restricted overlap and thus act almost as functional islands on the mainland. If this is true for other regions on the mainland, an intensified village-by-village malaria intervention might be more appropriate than was previously considered.

By genetic means such as microsatellites, it is possible to analyze meta-populations on a generational scale^22^. However, research on the spatial dynamics of mosquitoes over shorter time frames have required mark-release-recapture experiments where a large number of mosquitoes are radioactively labeled or marked with fluorescent dye before release. This technique is plagued by a plethora of technical problems and massive releases of vectors of disease bring ethical issues. For example, in Burkina Faso 21,000 *An. gambiae* females were released and only around 1% were recaptured in the same village and about 0.2% in the neighboring villages^23^. In contrast, by analyzing insect-bacterial associations, we are now able to study ecological distances within a much shorter time frame, using a limited number of specimens and without performing any releases. The generality of microbiotas as proxies for analysis of metapopulations and dispersal has large potential in animal research and shows that our lives are strongly interconnected with the bacteria surrounding us.

## Conclusion

In conclusion, this study shows that the microbiota from whole-body mosquitoes is mirroring the environment in which the mosquitoes live and that based on the microbiota, the mosquitoes can be assigned to different populations. The most important determinants for village connection represent three important life history events. The presence of *Wolbachia* reflects the locality of females (mothers) from the previous generation, *Shewanella* and *Massilia* sequences are probably obtained in breeding sites during the larval stage, and *Acinetobacter* sequences suggest that the nectar sources for the adults differ. However, in order to draw any strong conclusions, precise information about environment-specific bacteria is needed and thus will be the target for future studies.

## Methods

### Study sites and mosquito collection

**Figure 1:**
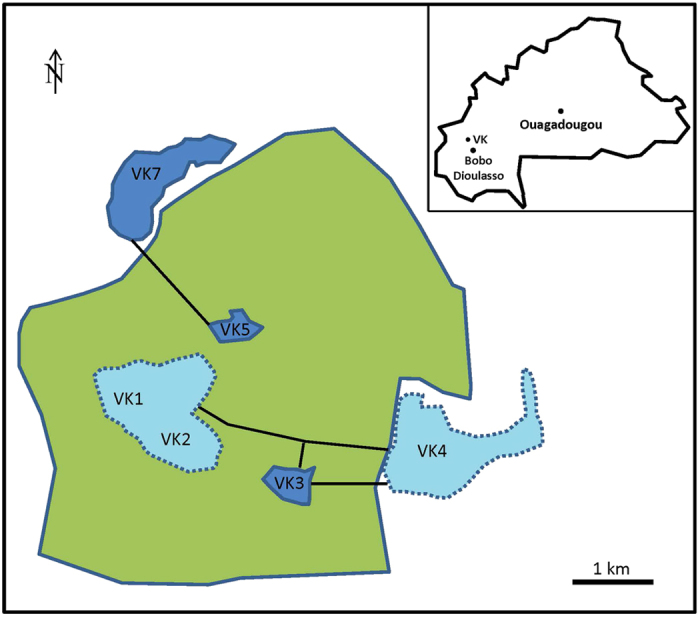
Locations of mosquito-collection villages. Rice field is marked in green. VK villages in dark blue are study sites. Black lines between villages indicate roads. The figure was made in Microsoft PowerPoint 2010.

The collection sites are located in the western part of Burkina Faso in the large rice-growing area Vallée du Kou situated about 30 km North-West of Bobo-Dioulasso (Fig. 1). The villages of VK3 and VK5 are surrounded by rice fields, while VK7 has rice fields in the South and Savannah in the North. Because of the irrigation system, rice fields form permanent mosquito breeding sites for the *An. gambiae* M form (*An. coluzzii*^24^). There is also maize, roots and mixed vegetable cropping in the area. Mosquitoes were collected inside houses on two consecutive days (15 and 16 May, 2012) with mouth aspiration and stored in individual eppendorf tubes until further processing. To avoid that mosquitoes contaminated each other, they were handled separately one at a time. From January to June, the *An. gambiae* local population is exclusively composed of the M form. We verified that the mosquitoes collected belonged to the *An. gambiae* M form by PCR-RFLP according to Fanello *et al.*^25^. In total, 60 mosquitoes were collected from three villages. To ensure enough bacterial DNA, the physically smaller males were pooled with five males each before DNA extraction and PCR. Thus a total of 36 samples were used.

### DNA and amplicon preparation

DNA from whole mosquitoes was prepared with the Promega Wizard Genomic DNA Purification Kit according to the manufacturer’s instructions. Bacterial 16 S rDNA (*Escherichia coli* position 341–805) were amplified by using general bacterial primers 341F (CCTACGGGNGGCWGCAG) and 805R (GACTACHVGGGTATCTAATCC)^26^. This primer pair matches approximately 90% of all good-quality bacterial sequences and covers all phyla in the Ribosomal Database Project release 10.25. Each DNA sample was individually PCR-amplified with Ready to go PCR beads (GE Health Care) by initial denaturation at 95 °C for 5 min followed by 35 cycles of [40 s at 95 °C, 40 s at 53 °C and 1 min at 72 °C] followed by a final 7-min extension at 72 °C. In a second PCR was added 1 of 50 flanking barcode sequence pairs to run samples in parallel^27^ using the same conditions as above, but only for 10 cycles of iteration.

### Library preparation

Sequencing libraries were prepared from 0.2 μg of PCR-product according to the TruSeq DNA sample preparation guide #15005180 revC using reagents from the TruSeq DNA sample prep kit set A and set B v2 (Illumina). Briefly, the DNA fragments were end-repaired followed by purification using AMPure XP beads (Beckman Coulter). An A-base was added to the blunt ends of the DNA fragments and adapters and index tags for sequencing were ligated, followed by purification using AMPure XP beads. The DNA-fragments were amplified for 10 cycles of PCR, followed by purification using AMPure XP beads (Beckman Coulter). The quality of the library was evaluated using the Fragment analyzer from Advanced Analyticals and the DNF-910 dsDNA reagent kit. The adapter-ligated fragments were quantified by qPCR using the Library quantification kit for Illumina (KAPA Biosystems) on a StepOnePlus instrument (Applied Biosystems/Life technologies) prior to cluster generation and sequencing.

### MiSeq sequencing

A 14 pM solution of DNA was subjected to cluster generation and paired-end sequencing with 300 bp read length on the MiSeq system (Illumina Inc.) using the v3 chemistry according to the manufacturer’s protocols. Base calling was done on the instrument by RTA 1.18.42 and the resulting .bcl files were demultiplexed and converted to fastq format with tools provided by CASAVA 1.8.4 (Illumina Inc.), allowing for one mismatch in the index sequence. Additional statistics on sequence quality were compiled with an in-house script from the fastq-files, RTA and CASAVA output files. Sequencing was performed by the SNP&SEQ Technology Platform in Uppsala, Sweden www.sequencing.se.

### Bioinformatics analysis

Sequences were processed using the illumitag pipeline as described in Sinclair *et al.*^27^. In short, the paired-end reads were merged using PANDAseq^28^, and filtered based on their Phred scores. Multiplexing-barcodes were cut-off, and chimeras removed. Chimeras and OTU (Operational Taxonomic Unit) clustering at 97% sequence similarity was done using UPARSE^29^. The taxonomical annotation of the found OTUs was performed by CREST using the SILVAmod-database^30^. A total of 1.8M reads were obtained and after processing^27^ it led to a final number of 670 k reads left for analysis. The sequences were distributed as shown in Supplementary Fig. S1. Based on the analysis of rarefaction curves (Supplementary Fig. S2), seven samples were removed from the analysis for having less than 7500 reads giving a final number of 560 k reads (on average 19,300 reads/sample). The remaining read counts for the remaining 29 samples were then rarefied to the lowest number of reads per sample (8452 for sample VK7_NBF1).

### NMDS plots and similarity heatmaps

Data analysis was done using the R-software (version 3.0.2). The heatmap trees were generated using the hierarchical clustering function from the R-software. The sample-wise horn-similarity heatmap was generated using the ‘vegdist’ function from the R-package ‘vegan’ (version 2.0–10), and the OTU- and taxonomical co-ocurrence heatmaps using simple pearson correlation. Non-metric dimensional scaling (NMDS) was performed using the ‘metaMDS’ function from the R-package ‘vegan’ and the horn’s dissimilarity index. Permutation-based ANOVA has been done with the ‘adonis’ function of the ‘vegan’ package with 10^6^ permutations.

### Predictive modeling

To provide predictive results of classification of mosquitoes according to their microbiome makeup, a supervised multivariate algorithm was developed using the R-software (version 3.2.0). In this algorithm, the ‘randomForest’ function from the ‘randomForest’ package (version 4.6–10) was used as classifying method within an in-house developed repeated double cross validation (rdCV) scheme^4^,^31^ to reduce the probability of statistical overfitting. Furthermore, the random forest was tuned over number of OTUs within the inner CV loop to provide an unbiased selection of the most informative OTUs, thereby optimising model performance^32^. In brief, tuning was performed by iteratively performing the inner validation loop using successively fewer OTUs, where in each step of the inner loop the 10% least informative OTUs (decided by average ranking of inner segment models) were removed. The double CV loop was repeated (n = 200) to achieve a population of class prediction probabilities per observation, thus enhancing the information content of prediction analysis. Permutation analysis was performed to test overall model validity and degree of overfitting by calculating the cumulative probability of actual model misclassification within a t-distributed H0 population. The H0 population was in turn populated by number of misclassification from models (n = 400) in which classifications labels were randomly drawn without replacement. Student’s t-distribution assumption was assessed by visually inspecting the H0 population histogram (Supplementary Fig. S4).

### *Wolbachia* 16 S rDNA amplification

For *Wolbachia* PCR detection on selected samples, primers specific for *Wolbachia* 16 S rDNA (W-SpecF 5′-CATACCTATTCGAAGGGATAG-3′ and W-SpecR 5′-AGCTTCGAGTGAAACCAATTC-3′) were used as for universal 16 S rDNA amplification with the following conditions: initial denaturation at 95 °C for 2 min followed by 2 cycles of [2 min at 95 °C, 1 min at 60 °C and 1 min at 72 °C] followed by 35 cycles of [30 s at 95 °C, 1 min at 60 °C and 45 s at 72 °C] followed by a final 5-min extension at 72 °C^33^. Positive bands were cloned and sequenced.

### Molecular phylogenetic analyses

**Figure 6:**
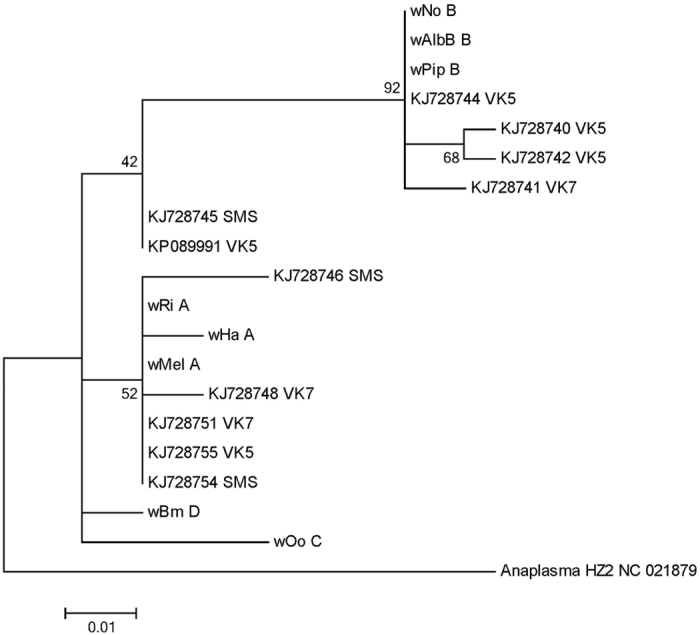
Molecular Phylogeny of *Wolbachia* WSPEC. Maximum Likelihood phylogenetic tree was conducted with 1000 bootstraps. The tree with the highest log likelihood (−300.4647) is shown. The percentage of trees in which the associated taxa clustered together is shown next to the branches. KJ sequences are from Baldini *et al.* (11); the KP sequence is from the current study. The location of each village is written after the GenBank sequence for the mosquito data. For the WSPEC from the *Wolbachia* genomes, the last capital letter denotes the *Wolbachia* supergroup.

The evolutionary histories of *Wolbachia* (and *Thorsellia*) were inferred by using the Maximum Likelihood method based on the Tamura-Nei model^34^ with 1000 bootstraps. The trees with the highest log likelihood (−300.4647 for *Wolbachia* and −1088.7983 for *Thorsellia*) are shown in Fig. 6 and Supplementary Fig. S6, respectively. The percentage of trees in which the associated taxa clustered together is shown next to the branches. Initial tree(s) for the heuristic search were obtained automatically by applying Neighbor-Join and BioNJ algorithms to a matrix of pairwise distances estimated using the Maximum Composite Likelihood (MCL) approach, and then selecting the topology with superior log likelihood value. A discrete Gamma distribution was used to model evolutionary rate differences among sites [5 categories (+*G*, parameter = 0.4473 for *Wolbachia*; +G, parameter = 0.1992, for *Thorsellia*)]. The trees are drawn to scale, with branch lengths measured in the number of substitutions per site. The analyses involved 20 *Wolbachia* nucleotide sequences and 10 *Thorsellia* nucleotide sequences, respectively. All positions containing gaps and missing data were eliminated. There were a total of 120 *Wolbachia* positions and 420 *Thorsellia* positions, respectively in the final datasets. Evolutionary analyses were conducted in MEGA6^35^.

## Additional Information

**How to cite this article**: Buck, M. *et al.* Bacterial associations reveal spatial population dynamics in *Anopheles gambiae* mosquitoes. *Sci. Rep.* **6**, 22806; doi: 10.1038/srep22806 (2016).

## Supplementary Material

Supplementary Information

Supplementary Data

## Figures and Tables

**Figure 2:**
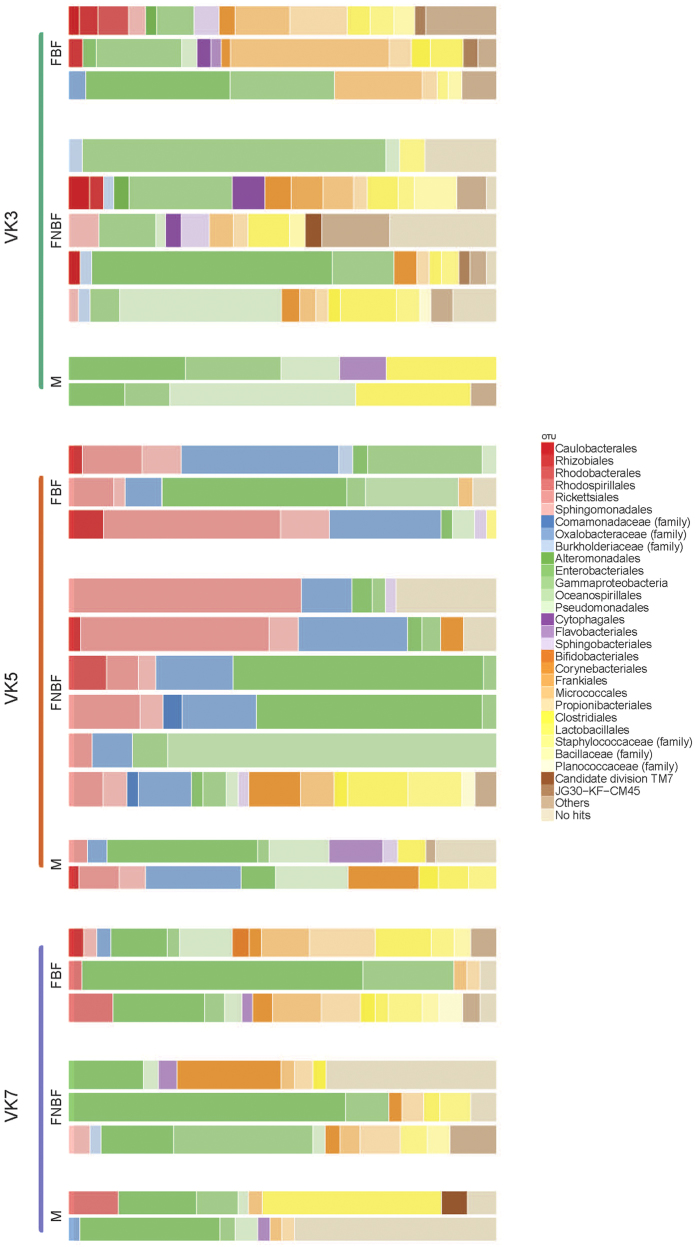
Taxonomic designation of microbiota from whole body of mosquitoes. Sequences are designated to order or families. M Male. FBF Female blood-fed. FNBF Female non-blood-fed.

**Figure 3:**
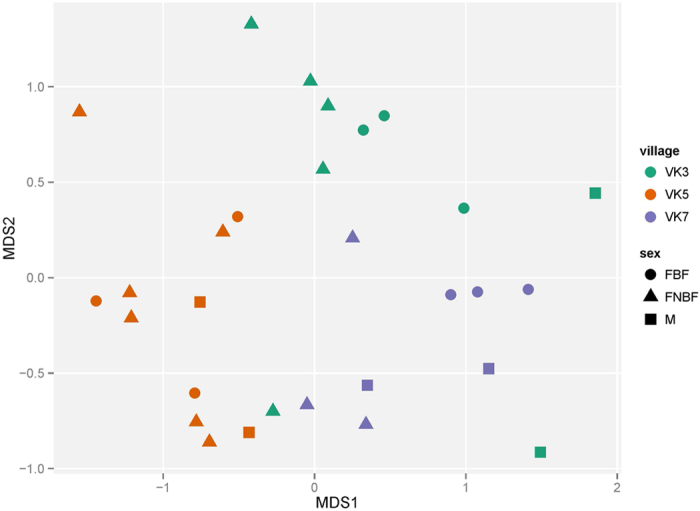
NMDS plot of all bacteria OTUs in *An. gambiae* mosquitoes. The mosquitoes clustered together have a more similar microbiota and show a strong correlation to village of origin. All OTUs of each sample are utilized for the clustering. The mosquitoes were collected from randomly selected houses in village VK5 at 15 May 2012 and in villages VK3 and VK7 at 16 May 2012. M Male. FBF Female blood-fed. FNBF Female non-blood-fed.

**Figure 4:**
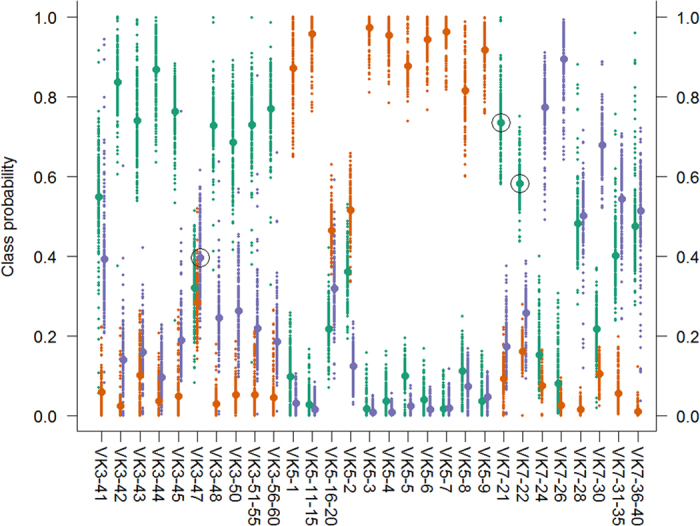
Predictive classification of mosquitoes (*An. gambiae*) according to village based on random forest modelling of OTU data. Multiple random forest modelling was performed within a repeated double cross validation scheme to minimize the risk of statistical overfitting (see method). Classification probability per submodel (n = 200) is color coded per village (VK3: green; VK5: orange; VK7: blue) and the global classification probability per individual and village marked in larger size and similar color. Successful classification was confirmed for 26 out of 29 individual samples (90%) and misclassified individuals are marked by a black circle. Of special interest are two individuals (VK7-21 and VK7-22) which although captured in VK7 possibly appertain to VK3 (p ≥ 0.58), thus indicating the potential to correctly identify migrating individuals.

**Figure 5:**
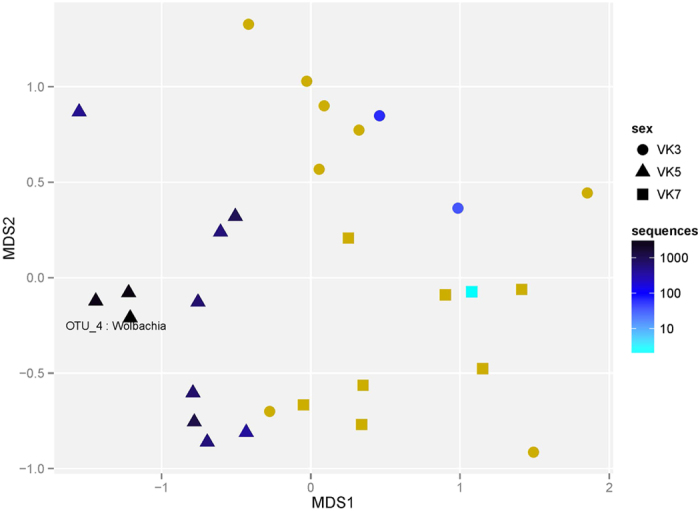
NMDS plot of all bacteria OTUs with color based on number of *Wolbachia* reads per sample. A high abundance of *Wolbachia* in village VK5 is seen. The positions of the samples are determined by the whole bacterial flora (identical to fig. 3), but the color is dependent on the number of *Wolbachia* sequences per sample. Yellow color indicates no sequences.

**Figure 7:**
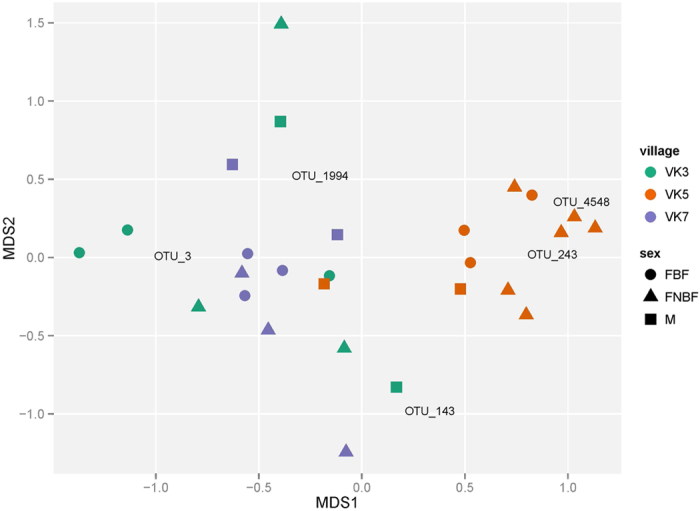
NMDS plot of *Acinetobacter* OTUs. The mosquitoes clustered together have a more similar microbiota and for VK5 show a strong correlation to village of origin. All OTUs belonging to *Acinetobacter* in each sample are utilized for the clustering. M Male. FBF Female blood-fed. FNBF Female non-blood-fed.
