# Supplementary material for: Bacterial associations reveal spatial population dynamics in Anopheles gambiae mosquitoes
Source: Sci Rep. 2016 Mar 10;6:22806. doi: 10.1038/srep22806 (PMC4785398; doi:10.1038/srep22806)
Supplement: Supplementary Information [file srep22806-s1.pdf]

SREP-15-25034A

Bacterial associations reveal spatial population dynamics in Anopheles gambiae mosquitoes

Moritz Buck, Louise K. J. Nilsson, Carl Brunius, Roch K. Dabire, Richard Hopkins, Olle Terenius

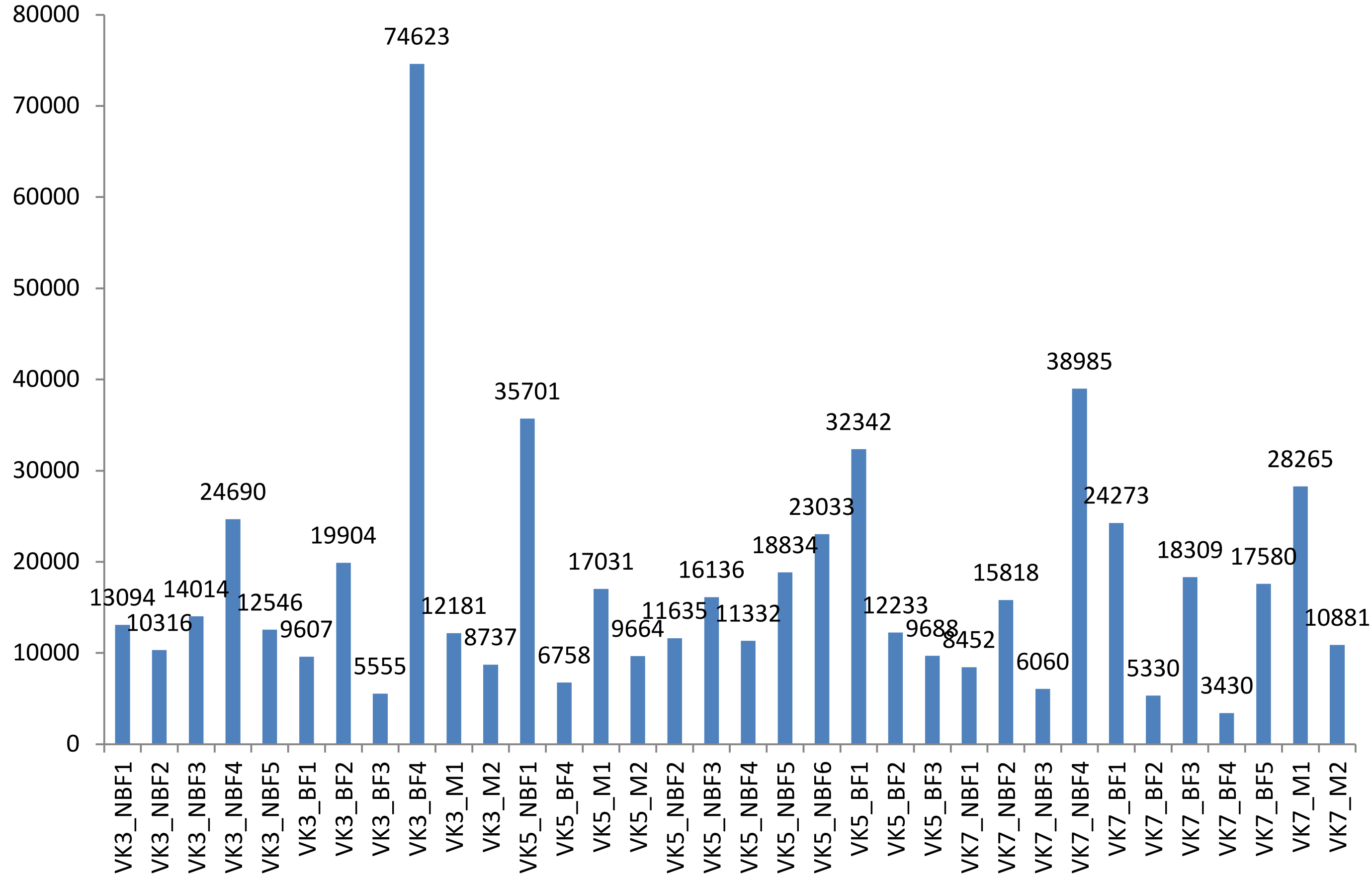

Supplementary Fig. S1. Number of sequences per sample before rarefaction analysis.

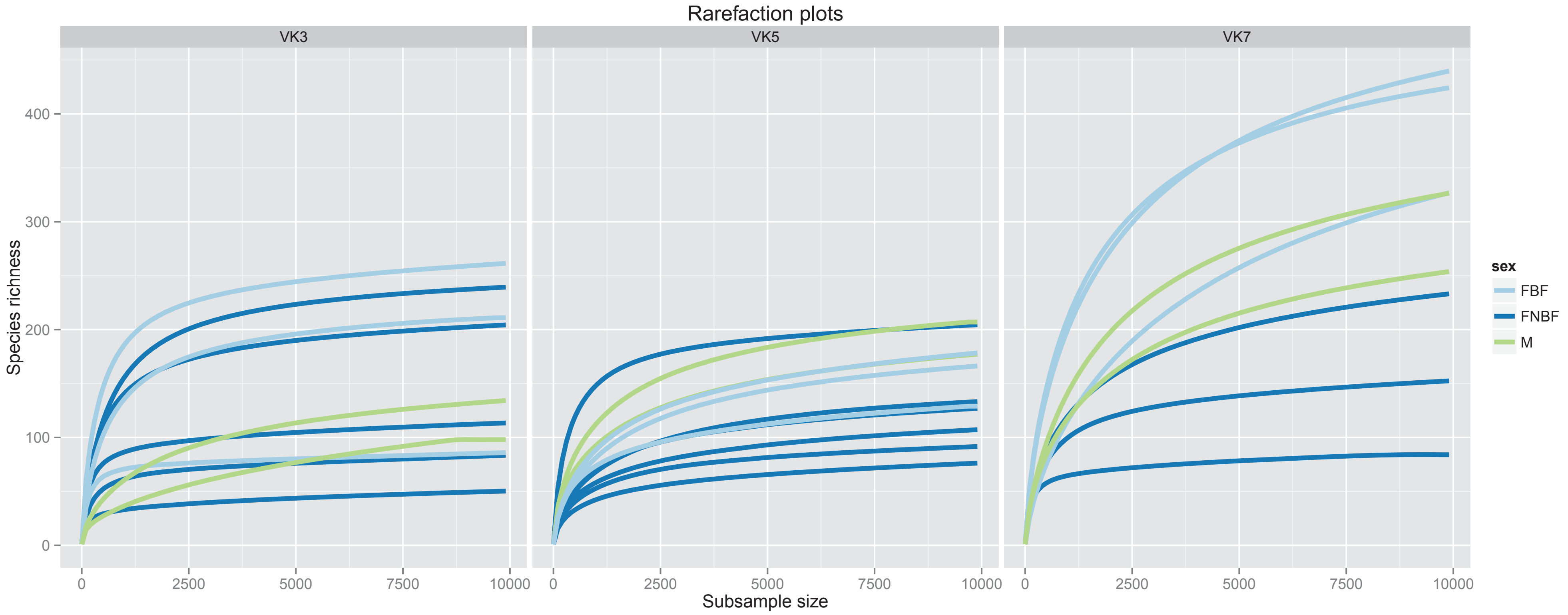

Supplementary Fig. S2. Rarefaction curves of number of reads per sample.

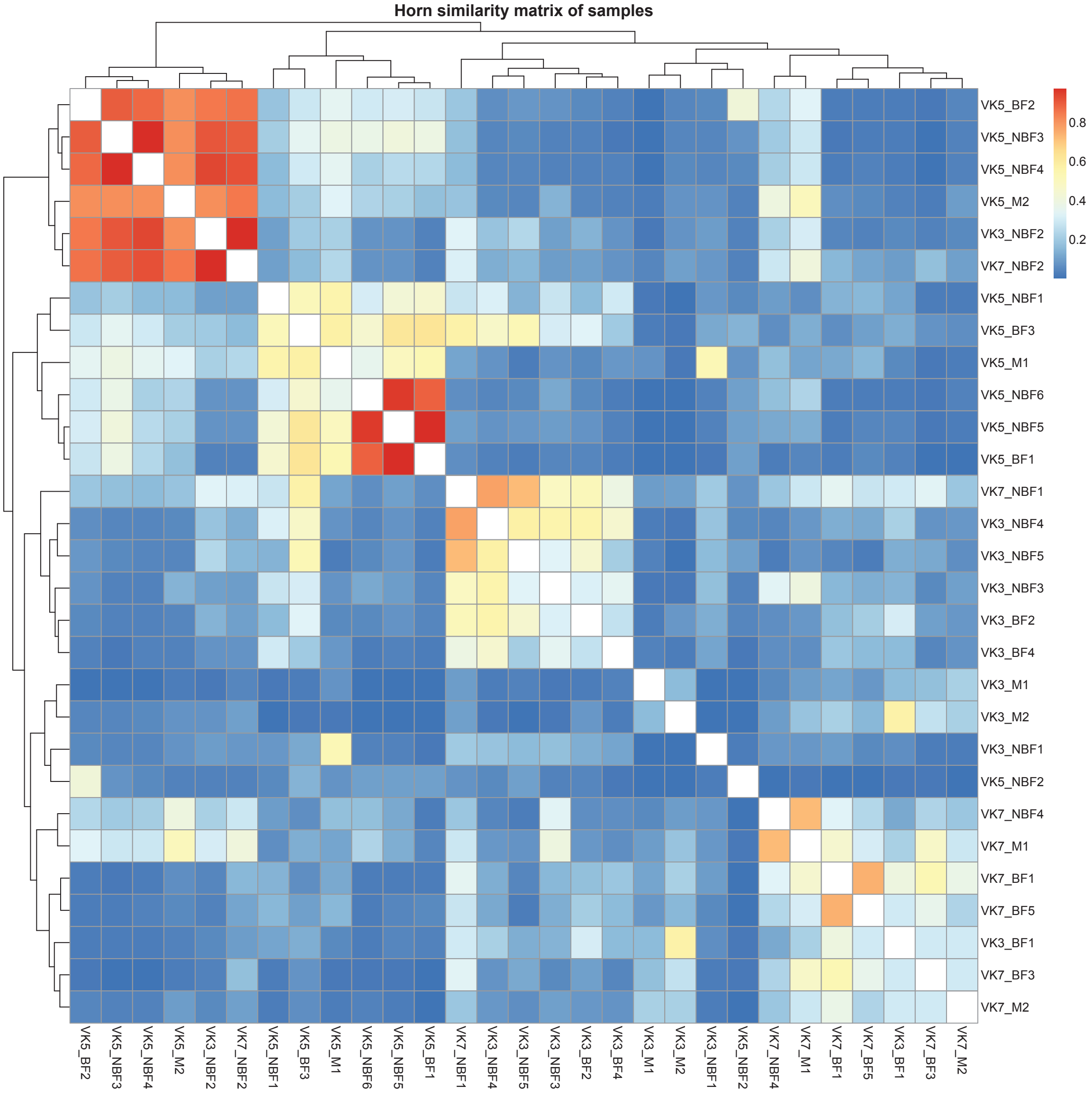

Supplementary Fig. S3. Horn similarity matrix of samples.

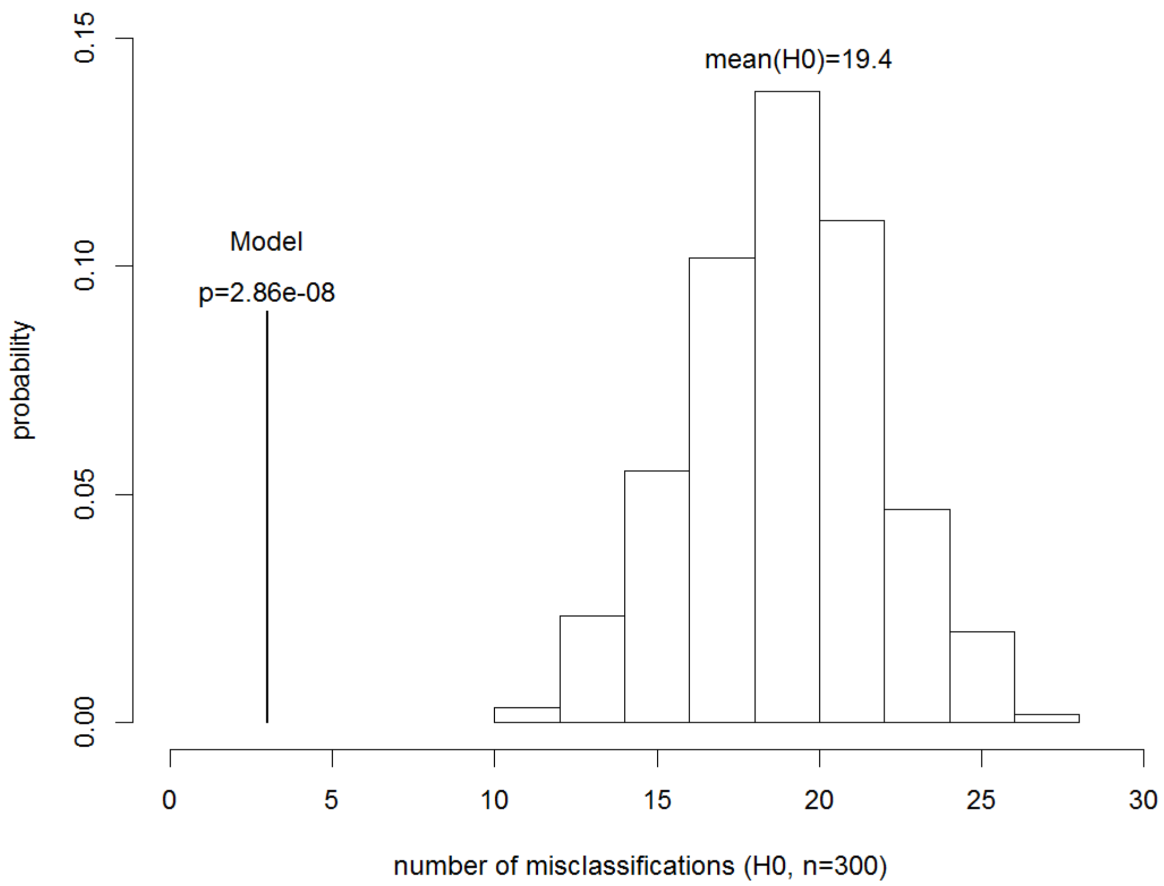

Supplementary Fig. S4 Permutation test of the repeated double cross-validated random forest (rdCV-RF) model for classification of mosquitoes (An. gambiae) according to village.

The actual model was compared to a H0 population (n=400) of rdCV-RF models of randomly attributed classification labels: The cumulative probability of the actual model (3 misclassifications) within the H0 t-distribution of permuted model misclassifications was p=2.4*10-8, indicating high model validity. Moreover, the H0 mean number of misclassification was 19.4 which, when compared to the expected E(n_misClass)=□(2/3 〖n〗_total)≈19.3, confirms that the general degree of overfitting using rdCV-RF modelling was negligible.

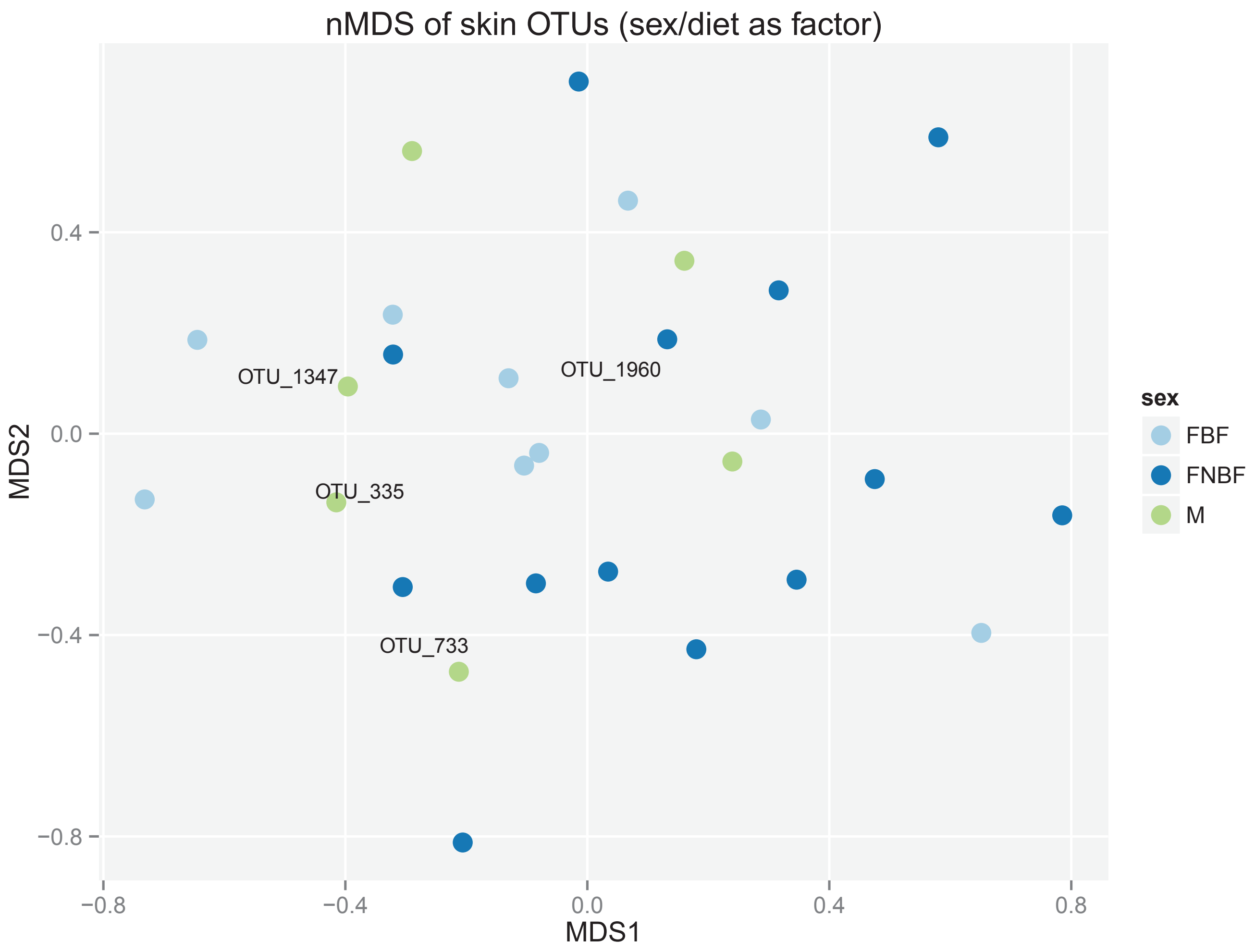

Supplementary Fig. S5. nMDS plots of OTUs of human skin bacteria Bacillus, Brevibacterium and Corynebacterium with sex/diet as determinant. These genera are the most attractive for female An. gambiae (4).

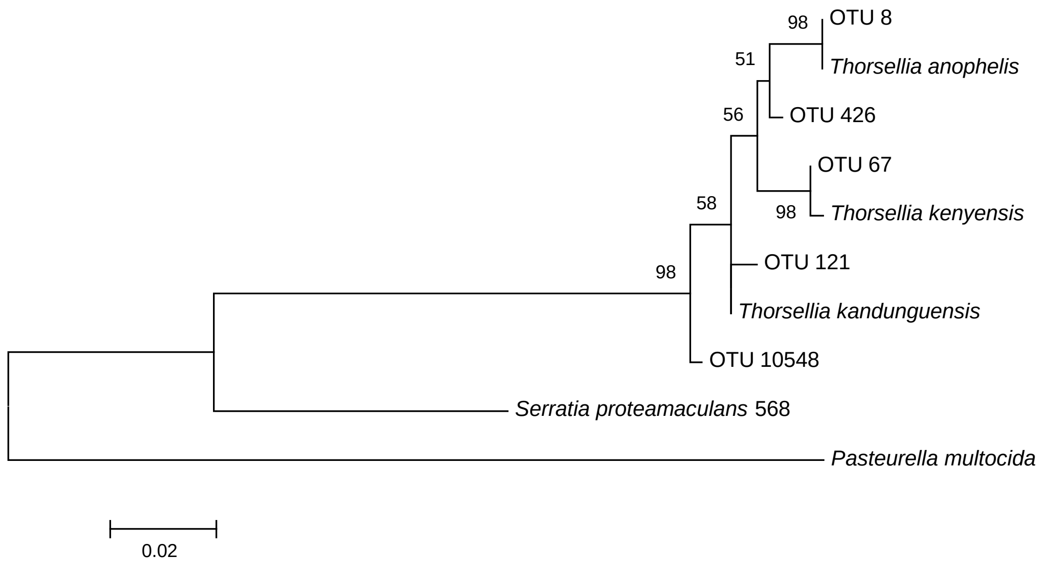

Supplementary Fig. S6. Molecular Phylogeny of Thorsellia 16S

The Maximum Likelihood phylogenetic tree was conducted with 1000 bootstraps. The tree with the highest log likelihood (-1088.7983) is shown. The percentage of trees in which the associated taxa clustered together is shown next to the branches.

Supplementary Table S1. Strongest determinants by Random Forest

OTU	Order	OTU	MeanRank	SD	CV	Taxon
28	1	OTU_28	2.3	0.2	10.6	Cellular organisms;Bacteria;Proteobacteria;Betaproteobacteria;Burkholderiales;Oxalobacteraceae;Massilia
4	2	OTU_4	7.1	25.5	357.0	Cellular organisms;Bacteria;Proteobacteria;Alphaproteobacteria;Rickettsiales;Anaplasmataceae;Wolbachia
400	3	OTU_400	26.2	64.3	245.0	Cellular organisms;Bacteria;Proteobacteria;Gammaproteobacteria;Alteromonadales;Shewanellaceae;Shewanella
3454	4	OTU_3454	40.0	79.5	198.5	Cellular organisms;Bacteria;Proteobacteria;Betaproteobacteria;Burkholderiales;Oxalobacteraceae;Massilia
243	5	OTU_243	47.8	96.0	200.8	Cellular organisms;Bacteria;Proteobacteria;Gammaproteobacteria;Pseudomonadales;Moraxellaceae;Acinetobacter
1208	6	OTU_1208	181.9	160.4	88.2	Cellular organisms;Bacteria;Proteobacteria;Gammaproteobacteria
178	7	OTU_178	194.0	168.0	86.6	Cellular organisms;Bacteria;Bacteroidetes;Flavobacteria;Flavobacteriales;Flavobacteriaceae;Wautersiella
114	8	OTU_114	203.3	164.8	81.0	Cellular organisms;Bacteria;Bacteroidetes;Sphingobacteria;Sphingobacteriales;Sphingobacteriaceae;Sphingobacterium
39	9	OTU_39	204.2	190.1	93.1	Cellular organisms;Bacteria;Proteobacteria;Gammaproteobacteria;Oceanospirillales;Halomonadaceae;Halomonas phoceae
16	10	OTU_16	208.3	181.0	86.9	Cellular organisms;Bacteria;Proteobacteria;Gammaproteobacteria;Enterobacteriales;Enterobacteriaceae
21	11	OTU_21	294.6	206.0	69.9	Cellular organisms;Bacteria;Proteobacteria;Alphaproteobacteria;Rhizobiales;Methylobacteriaceae;Methylobacterium
26	12	OTU_26	389.2	204.9	52.6	Cellular organisms;Bacteria;Actinobacteria;Actinobacteria (class);Propionibacteriales;Propionibacteriaceae;Propionibacterium
134	13	OTU_134	459.8	234.8	51.1	Cellular organisms;Bacteria;Proteobacteria;Betaproteobacteria;Burkholderiales;Comamonadaceae
1002	14	OTU_1002	568.6	218.5	38.4	Cellular organisms;Bacteria;Proteobacteria;Gammaproteobacteria;Pseudomonadales;Moraxellaceae;Acinetobacter
337	15	OTU_337	597.9	242.5	40.6	Cellular organisms;Bacteria;Proteobacteria;Betaproteobacteria;Burkholderiales;Oxalobacteraceae
99	16	OTU_99	675.6	224.1	33.2	Cellular organisms;Bacteria;Actinobacteria;Actinobacteria (class);Micrococcales;Microbacteriaceae

CV=RSD=SD/Mean
Coefficient of Variation = Relative Standard Deviation = Standard Deviation / Mean

Supplementary Table 2. Percentage of Acinetobacter per sample

VK3		VK5		VK7	
Sample	Acinetobacter	Sample	Acinetobacter	Sample	Acinetobacter
FBF1	1.1	FBF1	4.0	FBF1	2.5
FBF2	2.4	FBF2	0.7	FBF3	0.6
FBF4	0.1	FBF3	2.3	FBF5	8.5
FNBF1	2.0	FNBF1	1.9	FNBF1	1.3
FNBF2	0	FNBF2	0.3	FNBF2	0.3
FNBF3	1.3	FNBF3	0.5	FNBF4	1.4
FNBF4	0	FNBF4	0.4	M1	1.2
FNBF5	2.7	FNBF5	0.4	M2	1.7
M1	39.0	FNBF6	0.6		
M2	12.3	M1	3.0		
		M2	5.0		

FBF Female blood-fed

FNBF Female non-blood-fed

M Male
